# Trx2p-dependent Regulation of *Saccharomyces cerevisiae* Oxidative Stress Response by the Skn7p Transcription Factor under Respiring Conditions

**DOI:** 10.1371/journal.pone.0085404

**Published:** 2013-12-23

**Authors:** Rocío Gómez-Pastor, Elena Garre, Roberto Pérez-Torrado, Emilia Matallana

**Affiliations:** 1 Departament de Bioquímica i Biologia Molecular, Universitat de València, Valencia, Spain; 2 Departamento de Biotecnología, Instituto de Agroquímica y Tecnología de Alimentos, CSIC, 7 Paterna, Valencia, Spain; Kinki University School of Pharmaceutical Sciences, Japan

## Abstract

The whole genome analysis has demonstrated that wine yeasts undergo changes in promoter regions and variations in gene copy number, which make them different to lab strains and help them better adapt to stressful conditions during winemaking, where oxidative stress plays a critical role. Since cytoplasmic thioredoxin II, a small protein with thiol-disulphide oxidoreductase activity, has been seen to perform important functions under biomass propagation conditions of wine yeasts, we studied the involvement of Trx2p in the molecular regulation of the oxidative stress transcriptional response on these strains. In this study, we analyzed the expression levels of several oxidative stress-related genes regulated by either Yap1p or the co-operation between Yap1p and Skn7p. The results revealed a lowered expression for all the tested Skn7p dependent genes in a Trx2p-deficient strain and that Trx2p is essential for the oxidative stress response during respiratory metabolism in wine yeast. Additionally, activity of Yap1p and Skn7p dependent promoters by β-galactosidase assays clearly demonstrated that Skn7p-dependent promoter activation is affected by *TRX2* gene deficiency. Finally we showed that deleting the *TRX2* gene causes Skn7p hyperphosphorylation under oxidative stress conditions. We propose Trx2p to be a new positive efector in the regulation of the Skn7p transcription factor that controls phosphorylation events and, therefore, modulates the oxidative stress response in yeast.

## Introduction

The *TRX2* gene encodes the cytoplasmic thioredoxin II in *Saccharomyces cerevisiae*, which is a small protein (11 kDa) with thiol-disulfide oxidoreductase activity [1]. It was one of the first identified gene targets of Yap1p, the main oxidative stress transcriptional factor belonging to the Yap-βZIP (Yeast activator proteins) family [2,3], and it is also among the most highly induced genes in response to oxidative stress [4]. During yeast growth, the presence of at least one thioredoxin (*TRX1* or *TRX2*) is important for redox homeostasis maintenance [5]. However, Trx2p is more specialized in protection against ROS as sensitivity to H_2_O_2_ increases in the *trx2* but not in the *trx1* mutant [2]. 

Thioredoxins are involved in protein protection against oxidative and reductive stresses [6,7], and are responsible for the negative regulation of Yap1p activity [8]. *In vitro* analyses have indicated that the reduced form of Trx2p can also act as a reducing agent for Yap1p disulfide linkages, thus inactivating its transcription factor function [9]. Furthermore, they participate in the catalytic cycle of Orp1p (*GPX3*), which is a positive regulator of Yap1p activity [9–12]. Due to their oxidoreductase activity, thioredoxins can also regulate other proteins such as: (i) thioredoxin peroxidases (ii), 3´-phosphoadenosine 5´-phosphosulfate reductase (PAPS) [13], (iii) ribonucleotide reductase [14], (iiii) hexokinase II [15], and several proteins in *E. coli* and plants [16,17]. 

Under non stressed conditions, Yap1p exists in the cytoplasm and the nucleus, but it rapidly localizes only in the nucleus after oxidative stress [10,18] by activating many oxidative stress response (OSR) genes either itself [19,20] or by cooperation with other transcription factor Skn7p [21–24]. The Skn7p transcription factor constitutively localizes in the nucleus and regulates both osmotic and oxidative stress response gene expression [23,25]. However, the molecular mechanisms underlying these two regulatory functions differ. Skn7p activity under osmotic stress depends on the phosphorylation of the receiver domain aspartate, D427, by the Sln1p histidine kinase, whereas its activity under oxidative stress depends on serine/threonine (S/T) phosphorylation [23]. Oxidant-dependent Skn7p activation seems to be regulated by Yap1p as the strains lacking the Yap1p transcription factor do not show S/T Skn7p phosphorylation. It has been postulated that the oxidant-dependent phosphorylation of Skn7p is required to produce a strong association with Yap1p and an efficient transcriptional activation of several OSR genes [23]. However, very little is known about the molecular mechanism of Skn7p regulation under oxidative stress conditions [23,24]. 

The molecular model for oxidative stress regulation is still far from being solved and the role of thioredoxins on regulating Yap1p and Skn7p functions is difficult to assess if based on the phenotypes observed in different mutants, various studied strains and under treatment with distinct oxidant compounds. It is known that the transcriptional response differs for several reactive oxygen species and oxidant doses [11]. In addition, the nuclear localization of Yap1 *per se* does not ensure good tolerance to oxidative stress. For instance, by affecting the C-terminal region, which contains the nuclear export signal (NES), the constitutive nuclear localization of Yap1p increased tolerance to diamide, but caused hypersensitivity to H_2_O_2_ [11]. Furthermore, it has been recently published that H_2_O_2_ and diamide trigger Yap1p nuclear localization differently, therefore they promote distinct antioxidant responses [26]. Further evidence that supports the existence of different antioxidant transcriptional responses yet to be described is that Tsa1p deficiency alters the expression of several Yap1p-targeted genes (*TRX2, SOD2, CTT1*) in the presence of H_2_O_2_ without affecting Yap1p nuclear localization [27]. Hence, all these reports suggest that alternative mechanisms for the oxidative stress response still to be described may exist, which coordinate Trx2p, Tsa1p, Yap1p, Skn7p, and other putative regulatory proteins.

Studies into oxidative stress response regulation in *S. cerevisiae* have been carried out only with laboratory yeast strains mainly at low H_2_O_2_ doses. However, very little is known about the oxidative stress regulation in natural wine yeast strains, which are much more resistant than laboratory strains. Our wine strains studies done under industrial conditions in molasses medium have shown how the *TRX2* gene overexpression (T*TRX2*) increases biomass yield under respiratory conditions by not only improving the oxidative stress response, but also by preventing protein damages from carbonylation events [7,28,29]. Furthermore, a global transcriptional analysis of wine yeast strain under industrial conditions has demonstrated that *TRX2* gene manipulation affects the expression of several oxidative stress response genes during industrial performance [15]. Therefore, the comprehension of the molecular basis of oxidative stress in wine yeasts can help design new strategies to improve industrial processes. However, molasses are complex rich media with an unknown composition, a high sucrose concentration and other carbon sources, which make understanding the participation of Trx2p in oxidative stress response regulation very difficult.

In this study, we analyzed the involvement of Trx2p in the transcriptional response to oxidative stress at high H_2_O_2_ doses using different carbon source media in natural wine yeasts. We outline that Trx2p is involved mainly in the oxidative stress response under respiratory conditions by modulating Skn7p transcription factor activity by hyper-phosphorylation events to thus regulate the OSR gene expression.

## Materials and Methods

### Yeast strains and cultivation conditions

All the plasmids and *Saccharomyces cerevisiae* strains used in this study are described in Tables 1 and 2, respectively. We used the S. *cerevisiae* natural wine yeast strain T73 (CECT 1894) [30] as a yeast model. This strain has been previously modified to T73*ura3∆* [31] to construct other strains (Table 2) given the absence of auxotrophies in natural yeasts. All the modified yeast strains were obtained following the lithium acetate procedure as modified by [32]. 

**Table 1:** Plasmids used in this study.

**Plasmid**	**Reference**
pFA6a-13Myc-KanMX6	(Longtine et al., 1998)
*GCRE_GPX2_-CYC1_TATA_-lacZ*	(Tsuzi et al., 2004) From Dr Inoue
*YRE_TRX2_-CYC1_TATA_-lacZ*	(Gulshan et al., 2005) From Dr Moye-Rowley

**Table 2:** Strains used in this study.

**Strain**	**Description**	**Reference**
T73	WT	(Querol et al., 1992)
T73*ura3∆*	*URA3* gene disruption	(Puig et al., 1998)
T73*trx2∆*	*TRX2* gene deletion	(Gómez-Pastor et al., 2012)
T73*trx2∆ura3∆*	*TRX2* gene deletion and *URA3* gene disruption	(Gómez-Pastor et al., 2012)
T73-GCRE	*GCRE*_*GPX2*_ *-CYC1*_*TATA*_ *-lacZ*	This study
*trx2∆*-GCRE	*GCRE*_*GPX2*_ *-CYC1*_*TATA*_ *-lacZ*	This study
T73-pCEP12	*YRE_TRX2_-CYC1_TATA_-lacZ*	This study
*trx2∆*-pCEP12	*YRE*_*TRX2*_ *-CYC1*_*TATA*_ *-lacZ*	This study
T73-SKN7 (myc_13x_)**^*a*^**	SKN7-13Myc KanMX	This study
*trx2∆*-SKN7 (myc_13x_)	SKN7-13Myc KanMX	This study

^a^ myc13x, thirteen copies of the myc epitope tag

Plasmid YRE_TRX2_-CYC1_TATA_-lacZ (pCEP12) contains a modified *TRX2* promoter regulated by Yap1p [26]. This plasmid was constructed by subcloning the region from -181 to -155 corresponding to the YRE sequence of the *TRX2* promoter into BglII-digested p314ClZ containing CYC1_TATA_-lacZ [26] for Yap1p promoter recognition. Plasmid GCRE_GPX2_-CYC1_TATA_-lacZ contains a modified *GPX2* promoter regulated by the combined action of Yap1p and Skn7p. This plasmid was constructed using the region from -284 to -269 of the *GPX2* promoter, which contains only the GCRE sequence (GC Rich Element) that is essential for Skn7p promoter recognition [33]. The PCR product was subcloned into the XhoI site of pTBA30, which contains CYC1_TATA_-lacZ that lacks its original upstream activation site (UAS) [33] for Skn7p-Yap1p recognition. Both plasmids have an *URA3* gene as a selectable marker.

To construct the SKN7-(myc_13x_) strains, complementary oligonucleotides to the tagging cassette (pFA6a-13Myc-KanMX6) were fused to the 3´end sequence of the target open reading frame with no stop codon (underlined sequence) for C-terminal tagging by PCR (F1; GCAATTACCACAATCTACACTTCAAGAAAACCAGCTATCACGGATCCCCGGGTTAATTAA and R2; GTCCTCTGCTAACTTAGACGCAAGGCTATTTGTAAAATTGAATTCGAGCTCGTTATAAAC), as described by Longtine et al., [34]. The amplified PCR product was used for yeast modification by homologous recombination.

For all experiments, cells were cultivated previously in SD minimal medium (2% glucose, 0.5% ammonium sulfate, 0.15% yeast nitrogen base) and a mixture of amino acids at 30°C, and were then inoculated in YPD (1% yeast extract, 2% bacto-peptone, 2% glucose) or YPG (1% yeast extract, 2% bacto-peptone, 2% glycerol) media. To test sensitivity to different oxidant compounds, cells from an overnight culture were diluted to 1.0 OD_600_ nm and spotted on YPD and YPG plates containing 5 mM H_2_O_2_, 0.1 mM menadione, 1.2 mM diamide or 16.5% ethanol, and plates were incubated between 3 and 5 days. The oxidative stress experiments in liquid were carried out by adding different H_2_O_2_ concentrations (range 0.4-5 mM) for 1 h at 30°C to the culture growth in the mid-log phase. Afterward, cells were harvested by centrifugation and frozen in N_2_ liquid. For the viability assays, cells were exposed to different H_2_O_2_ concentrations (0.4-5.0 mM) for 1 h and were then plated onto YPD or YPG medium. The number of viable cells was counted after 2 days at 30°C.

### Catalase activity determination

The cell extracts employed for enzymatic determination were prepared using glass beads and were assayed as described for catalase [35]. Catalase activity was assayed spectrophotometrically by adding 10-50 µg of protein sample to 0.2 mL of 50 mM phosphate buffer pH 7.0 and 80 mM H_2_O_2_. The decrease in absorbance at 240 nm due to H_2_O_2_ consumption was measured and enzyme activity was calculated using an extinction coefficient of 43.66 M^-1^cm^-1^. Catalase activity was expressed as µmol of H_2_O_2_ min^-1^ mg of protein^-1^ (U mg prot^-1^). Three independent experiments were done.

### Quantification of lipid peroxidation

Quantification of lipid peroxidation was carried out in a reaction of thiobarbituric acid with the malondialdehide (MDA) product of the oxidized fatty acid breakage [36]. Cells (50 mg) were collected, washed twice with distilled water and then extracted by vortexing with 0.3 g glass beads in 0.5 ml of 50 mM sodium phosphate buffer, pH 6.0, 10% trichloroacetic acid (TCA), in three 1-minute series alternated with a 1-minute incubation on ice. After centrifugation at 13000 rpm for 10 min, 300 µl of the supernatants were mixed to 100 µl of 0.1 M EDTA and 600 µl 1% thiobarbituric acid (Sigma Aldrich Co., St. Louis, MO) in 0.05 M NaOH, which were then incubated at 100°C for 15 min. After cooling on ice and centrifugation to eliminate precipitates, MDA was measured by reading absorbance at 535 nm. The molar absorptivity of MDA (1.56 ×10^5^ M^-1^ cm^-1^) was used to express the lipid peroxidation levels as pmoles of MDA per mg of cells.

### Glutathione determination

Glutathione was determined as previously described [37]. Collected cells (100 mg) were washed twice with phosphate-buffer saline (PBS pH 7.4) and suspended in 1 ml ice-cold 8 mM HCl, 1.3% (w/v) 5-sulphosalicylic acid (Fluka-Chemika, Switzerland). Cells were broken in Fast Prep at 4°C with 0.5 g of glass beads in three series of 30 s alternated with a 1-minute incubation on ice. All material-like tips and Eppendorf tubes were kept cold during glutathione extraction. Cell debris and proteins were pelleted in a microcentrifuge for 15 min (13000 rpm at 4°C), and supernatants were used for glutathione determination. For total glutathione determination, the supernatant was used directly in 200 µl of total volume reaction, while oxidized glutathione (GSSG) determination was carried out in the same volume reaction with 2 µl of 1 M 2-vinyl-piridine (Sigma Aldrich Co., St. Louis, MO, USA) for 1 h at room temperature. All the samples were incubated in the darkness with the enzyme cocktail (glutathione reductase 1.92 U/mL, glucose-6-phosphate dehydrogenase 0.125 mg/mL, NAPDH 16 mg/mL, glucose-6-phosphate 6.4 mg/mL) for 20 min at room temperature and shaking 125 rpm in the presence of 200 µM of 5, 5'-dithio-bis-(2-nitrobenzoic acid). Reduced glutathione level (GSH) was obtained as the difference between total glutathione and oxidized glutathione (GSSG). The positive control samples for total glutathione determination were fortified with different concentrations of GSH. The negative control samples for the GSSG assay were treated with 0.1 mM DTT prior to 2-vinyl-piridine. Finally an aliquot of each sample was treated with only phosphate buffer under the same conditions as described above for glutathione determination and were then used as an internal absorbance control. Data were expressed as the ratio between the GSH/GSSG levels.

### Analysis and quantification of mRNA

Total yeast RNA was obtained from yeast cells (50 mg) by two methods. In the antioxidant gene analysis, RNA was obtained by the hot phenol method [38]. For the Heat Shock Proteins (HSPs) mRNA analysis, total RNA was obtained by resuspending cells in LETS buffer (200 mm LiCl, 20 mm EDTA, 20 mm Tris-HCl (pH 8.0), 0.4% SDS), and was transferred to an Eppendorf screw-cap tube containing 0.5 ml of phenol and 0.5 ml of glass beads (acid-washed, 0.4 mm diameter). Equal amounts of RNA (20 µg) were separated in 1% (w/v) agarose gels containing formaldehyde (2.5% v/v) and were transferred to a Hybond nylon membrane (Amersham Biotech, GE Healthcare, Germany). The specific primers for the PCR synthesis of the DNA probes are provided in Table S1. The probes for the antioxidant gene analysis were labeled by random priming (High Prime, Roche Diagnostics, Indianapolis, IN) using [α^32^P]-dCTP (Amersham Biotech, GE Healthcare, Germany). For the *GLR1, GTT1* and *TRX1* genes, further mRNA quantification was performed by a qPCR analysis using RNeasy kit (Quiagen) for RNA extraction. Primers used for qPCR analysis (*GTT1*-F: GTCCATTGGTTGGCCATTC, *GTT1*-R: TGCAATATCAGCATCTTCGC, *GLR1*-F: ATTTTCCCCGAAAACATTCC, *GLR1*-R: AATTACCAAGTGCGTTTCGG, *TRX1*-F: AGCGAATTCGACTCTGCAAT, *TRX1*-R: TTGTGCAACATCACCCAAT). Obtained data was normalized using Actin (*ACT1*-F: TGTCACCAACTGGGACGATA, *ACT1*-R: AACCAGCGTAAATTGGAACG) as a control and normalized to control conditions in YPD.

The HSPs analysis probes were labeled with the non-radioactive PCR digoxigenin probe synthesis kit (Roche Diagnostics, Indianapolis, IN). Membrane pre-hybridizations and hybridizations were also performed with Digoxigenin Easy Hyb solution (Roche Diagnostics, Indianapolis, IN). After two stringent washes with 2X SSC (3 M NaCl, 0.3 M sodium citrate) 0.1% (w/v) SDS for 15 min at 65°C and 25°C, respectively, blots were subjected to immunological detection using an anti-digoxigenin antibody conjugated with alkaline phosphatase (Roche Diagnostics, Indianapolis, IN, USA), followed by CDP-Star detection (Roche Diagnostics, Indianapolis, IN). Images were captured with the LAS-1000 Plus imaging system (Fuji, Kyoto, Japan). For the radioactive membranes, images were captured and quantified with an Instant Imager FLA-5000 and the Image Gauge software (FujiFilm, USA). Sample data were normalized in relation to rRNA. An internal control gene was used for the consecutive membrane hybridizations. Gene expression experiments were performed in triplicate.

### The β-galactosidase assay

Cells were cultured in YPD or YPG medium until the log phase and were treated with H_2_O_2_ for 1 h. The cell extracts and assays of β-galactosidase activity were prepared as described by Miller [39]. One unit of activity was defined as the amount of enzyme that increased the OD_420_ by 1000 per hour at 30°C. Protein concentration was determined in a Nanodrop ND-1000 UV/Vis Spectrophotometer.

### Western blot analysis of Skn7p phosphorylation

Trichloroacetic acid (TCA) extracts were prepared from the cultures harvested in the log phase and were treated, where indicated, with H_2_O_2_ (0.4 and 5mM) for 1 h. Cell pellets were frozen and were then disrupted by vortexing in water and glass beads. TCA was added to 20% and 10 cycles of a 30-second vortex and 1-minute incubation on ice were performed. After adding 1 ml ice-cold 5% TCA, samples were centrifuged at high speed for 15 min at 4°C. The protein pellet was washed twice with cold acetone and was allowed to dry at room temperature for 5 min. Protein pellets were resuspended in protein buffer (20 mM HEPES, pH 8.0, 5 mM EDTA, 20% glycerol, 7 mM β-mercaptoethanol, and a mix of protease inhibitors (Roche Diagnostics, Indianapolis, IN, USA). Lambda phosphatase (New England Biolabs Gmbh, Frankfurt, Germany) was added to 100 U and samples were incubated for 30 min at 30°C. When required, 1X Phosphatase inhibitor cocktail 3 (Sigma Aldrich Co., St. Louis, MO) was also added before incubation. Samples were subjected to 10% SDS-polyacrylamide gel electrophoresis (SDS-PAGE) for 2.5 h at 200 V. Separated proteins were transferred onto PVDF membranes (Amersham Biotech, GE Healthcare, Germany). The Skn7-Myc fusion protein expression was examined by an immunoblot analysis with an anti-Myc antibody [40] followed by a goat anti-mouse secondary antibody (1:2.500; Amersham Biotech, GE Healthcare, Germany). The immune complexes were visualized by chemiluminescence using the Las1000 software (Fujifilm, USA) and the ECL Advance Detection Kit (Amersham Biotech, GE Healthcare, Germany).

### Immunoprecipitation

Cell lysates were prepared in Urea buffer (50 mM Tris-HCl [pH 8], 5 mM EDTA, 6 M urea, 1% SDS) and a mixture of commercial proteases (Roche Diagnostics, Indianapolis, IN, USA). Cells were disrupted with glass beads at 4°C. The obtained lysates were cleared by centrifugation and normalized to an equal amount of protein by a DC protein assay (Bio-Rad). Cell lysates were diluted with a 10x volume of immunoprecipitation (IP) buffer (50 mM Tris-HCl [pH 8], 5 mM EDTA, 1% Triton X-100, 150 mM NaCl) and a mixture of protease and phosphatase inhibitors. Lysates were rotated overnight at 4°C with anti-Myc agarose beads (A7470; Sigma). After washing with IP buffer 3 times, beads were incubated with SDS sample buffer for 30 min at 37°C and protein samples were subjected to SDS-PAGE. The antibodies used for the Skn7p post-translational modification analysis were anti-phosphoserine (Abcam, ab9332), anti acetyl-lysine (Biomol SA-440) and anti-sumo1 (Abcam ab32058).

## Results

### Effects of TRX2 gene manipulation and growth medium on the oxidative stress response

Recent studies have shown that laboratory strains are genetically very distant to any other *S. cerevisiae* strains and suggest the use of strains from a different source to study yeast physiology [41,42]. Thus we selected T73 for our study, a widely used wine yeast model strain, and we analyzed the effects of *TRX2* gene deficiency on the oxidative stress response. We used a defined medium under either fermenting conditions with glucose as a carbon source (YPD) or respiratory conditions in glycerol (YPG). 

**Figure 1:**
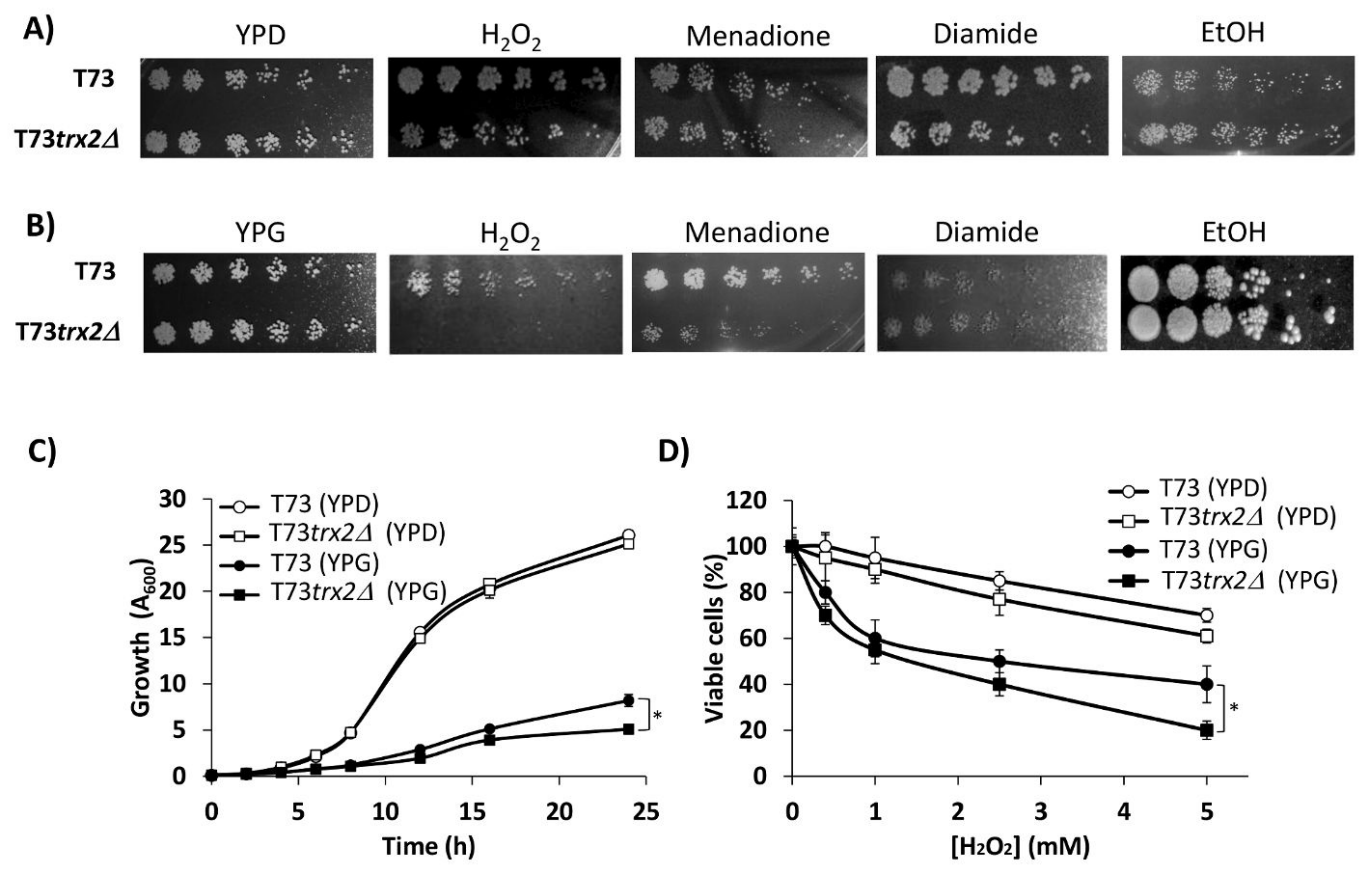
Comparison of the sensitivity of strains T73 and T73*trx2Δ* to different stressful conditions. Growth of strains T73 and T73*trx2∆* indicated on YPD (A) or YPG plates (B) containing no chemicals as a control, 5 mM H_2_O_2_, 0.1 mM menadione, 1.2 diamide, 16.5 % ethanol. For each strain, 5 µL of the overnight culture diluted to 1.0 OD_600nm_ units and seven subsequent dilutions (10^-1^ dilution factor) were spotted on plates. Growth was monitored after 2 days for YPD plates and 3 days for YPG plates. (C) The growth rate for strains T73 and T73*trx2∆* over 25 h in distinct carbon source media. (D) Viable cells after 1 h exposure to different H_2_O_2_ doses in various media. Three independent experiments were carried in all conditions.

We tested different oxidative stress conditions in T73 and T73*trx2∆* in either YPD or YPG medium (Figure 1). As seen in Figure 1A the absence of *TRX2* gene showed no significant differences when compared with the control strain in YPD. In fact, both strains showed high resistance to all the tested conditions in high glucose media. However, when cells were spotted on YPG (Figure 1B) and were grown under respiratory conditions, the absence of the *TRX2* gene dramatically increased sensitivity to H_2_O_2_ and menadione, although no differences in diamide and only a slight decrease in sensitivity to ethanol were observed. Afterward, we focused our experiments on analyzing H_2_O_2_ toxicity in wine strains as a common oxidant compound. When we analyzed the growth rate in the different carbon source media for 24 h (Figure 1C), we observed that the T73*trx2∆* mutant showed similar growth to the control strain in YPD, but that it significantly lowered in YPG medium at 24 h (p = 0.00197). The number of viable cells after a 1-hour exposure to different H_2_O_2_ concentrations (Figure 1D) also demonstrated that Trx2p deficiency was more important at a high H_2_O_2_ dose (2.5-5 mM) under respiratory conditions. These results reveal the involvement of Trx2p in the oxidative stress response in wine yeasts under respiratory conditions at a high H_2_O_2_ dose. 

To test the effect of Trx2p deficiency on the cellular redox state, we analyzed some oxidative-stress biochemical markers, such as catalase activity, lipid peroxidation and the glutathione levels in the exponentially grown cells of strains T73 and T73*trx2∆* after exposure to 5 mM H_2_O_2_ for 60 min (Table 3). The results obtained in YPD medium revealed that catalase activity was greater in the T73*trx2∆* strain than in the T73 strain. After H_2_O_2_ addition, catalase activity increased in both strains, but was significantly higher in T73*trx2∆* than in T73. Conversely, catalase activity in YPG medium was significantly lower in T73*trx2∆* than in T73 after H_2_O_2_ addition, which could explain the deficiency in growth rate observed for T73*trx2∆* under fermenting conditions. However no significant differences were observed in the lipid peroxidation levels and the GSH/GSSG ratio under either the control or stressful conditions in any carbon source media. 

**Table 3:** Biochemical redox parameters for wine yeast strains.

	**Catalase activity (U/mg prot)**				**Lipid peroxidation (pmol/mg prot)**				**Ratio GSH/GSSG**			
	T73		T73*trx2∆*		T73		T73*trx2∆*		T73		T73*trx2∆*	
**YPD**												
Control	5.12±0.85		7.12±0.85 *		12.21±0.29		11.85±0.52		55.1±5.2		48.3±4.1	
H_2_O_2_	6.60±0.86		10.18±1.7 *		17.60±2.68		21.19±0.48		31.0±5.4		23.1±6.2	
**YPG**												
Control	93.17±5.27		86.91±6.52*		3.15±0.69		3.48±0.35		17.5±2.3		15.2±1.7	
H_2_O_2_	107.99±5.13		79.59±4.51*		11.36±1.94		10.92±2.46		15.0±2.1		14.3±4.4	

Experiments were carried out in triplicate and presented error corresponds to SD. Statistical analyses were performed using a Student´s t-test between samples in the same growth media, YPD or YPG (* p< 0.05).

### Expression of the Yap1p-Skn7p-regulated antioxidant genes is dependent on the presence of TRX2 under respiratory conditions

**Figure 2:**
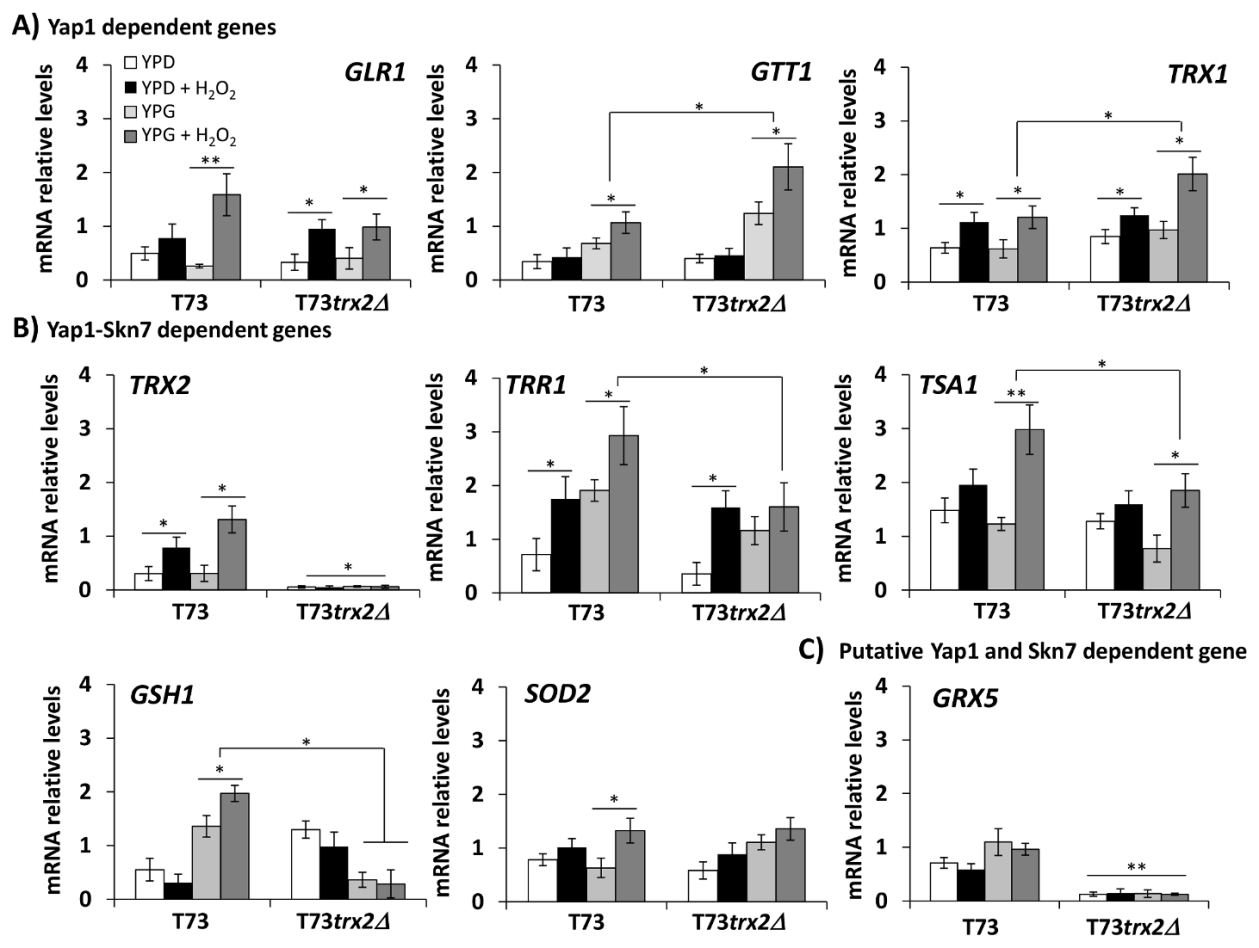
Antioxidant gene expression levels in strains T73 and T73*trx2∆* cultured in YPD or YPG medium to the mid-log phase and treated with 5 mM H_2_O_2_ for 60 min. The relative expression levels of each antioxidant gene were quantified from the Northern image analysis using the Image Gauge software (FujiFilm, USA). The data for each gene were normalized to the rRNA levels. Experiments were carried out in triplicate and the error bars corresponding to SD are shown. Statistical analyses were performed using a Student´s *t*-test between samples in the same growth media, YPD or YPG (* p< 0.05, ** p< 0.01). The Yap1p and the Yap1p-Skn7p-dependent gene groups were formed according to the previously described information [21, 22, 43] and from the Yeastract web site (http://www.yeastract.com).

It has been reported for laboratory yeast strains that thioredoxins are negative regulators of the main antioxidant transcription factor Yap1p as it is constitutively active in the double mutant *trx1∆trx2∆* [8]. However, the absence of one thioredoxin does not seem to alter the gene expression in laboratory yeast strains, although it does in a T73*trx2∆* strain under industrial propagation conditions in molasses medium [15]. In addition, very little is known about the regulatory mechanisms controlling other important transcription factors like Skn7p, which co-operate with Yap1p to better respond to oxidative stress. Thus, we evaluated the influence of *TRX2* gene manipulation on the expression of a set of selected antioxidant genes regulated by either Yap1 or the co-operation between Yap1 and Skn7 under oxidative stress conditions using different carbon source media. Quantification of the mRNA levels from the northern signals (Figure S1) of three independent experiments is shown in Figure 2. We selected a set of genes belonging to the glutathione-glutaredoxin system (*GSH1, GLR1, GTT1* and *GRX5*), to the thioredoxin system (*TRX1, TRX2, TRR1* and *TSA1*) and other gene coding for the antioxidant enzyme superoxide dismutase, *SOD2*. Among all the selected genes, some were regulated by Yap1p alone (*GLR1, GTT1* and *TRX1*) and others jointly by Yap1p and Skn7p (*GSH1*, *TSA1*, *TRR1*, *TRX2* and *SOD2*), as previously described [21,22,43].

Figure 2A shows the mRNA expression levels for the Yap1p-dependent genes for strains T73 and T73*trx2∆*. We observed that strain T73*trx2∆* displayed significantly higher expression levels for *GTT1* and *TRX1* in YPG medium after exposure to 5 mM H_2_O_2_, but no differences were observed in YPD as compared with the control strain. The gene expression for *GTT1, GLR1* and *TRX1* was validated by a qPCR analysis, which obtained similar results (Figure S2). These data indicate that the absence of Trx2p can activate Yap1p-dependent genes, but only under respiratory conditions and in the presence of H_2_O_2_, and then that Trx2p might be involved in negative regulation of Yap1p activity under those conditions. 

When we analyzed the expression levels for the Yap1p-Skn7p-dependent genes (Figure 2B), we observed the opposite effect as T73*trx2∆* showed a significant decrease in the mRNA expression levels for genes *TRR1*, *TSA1* and *GSH1* in YPG medium plus H_2_O_2_. In addition, the putative Yap1p and Skn7p-dependent *GRX5* gene expression also significantly lowered in T73*trx2∆* under all the tested conditions (Figure 2C). All these results suggest an involvement of Trx2p in Skn7p regulation activity, especially under respiratory conditions.

### Trx2p is involved in the promoter regulation by the Skn7p transcription factor

**Figure 3:**
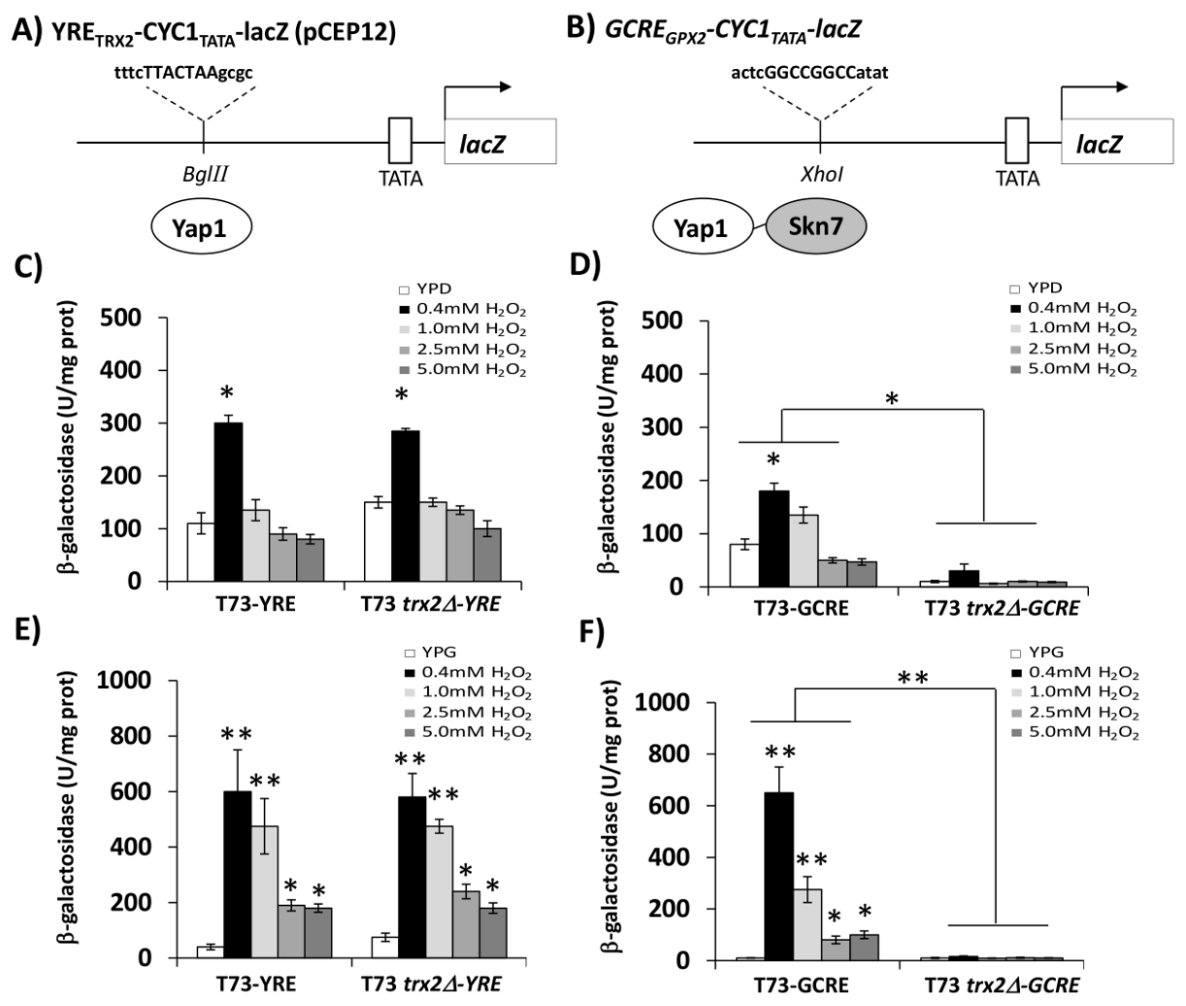
Promoter regulation by the Yap1p and Skn7p transcriptional factors in strains T73 and T73*trx2∆*. (A) The cells carrying the depicted modification of the TRX2 promoter fused to the LacZ vector construction (T73-YRE and T73*trx2∆*-YRE) for Yap1p recognition or (B) the cells carrying the depicted modification of the GPX2 promoter fused to the LacZ vector construction (T73-GCRE and T73 *trx2∆*-GCRE) for Yap1p-Skn7p recognition were cultured in YPD (C, D) or YPG medium (E, F) until the mid-log phase and were treated with different H_2_O_2_ concentrations (0.4, 1, 2.5 and 5 mM) for 60 min. Experiments were carried out using three biological replicates and the error bars corresponding to SD are shown. Statistical analyses were performed using a Student´s *t*-test between samples in the same growth media, YPD or YPG (* p< 0.05, ** p< 0.01).

To study the involvement of *TRX2* in Skn7p-mediated transcriptional regulation, we used two different plasmid constructs (Figure 3A and 3B), both containing the *URA3* auxotrophic marker. Plasmid YRE_TRX2_-CYC1_TATA_-lacZ (pCEP12) contains a modified *TRX2* promoter regulated by Yap1p, whereas plasmid GCRE_GPX2_-CYC1_TATA_-lacZ contains a modified *GPX2* promoter regulated by the combined action of Yap1p and Skn7p. 

Strains T73*ura3∆* and T73*trx2∆ura3∆* were transformed with both plasmids separately to give strains T73-YRE and T73-GCRE, and strains T73*trx2∆*-YRE and T73*trx2∆*-GCRE. The cells from these strains were exponentially grown in YPD and YPG media at different H_2_O_2_ concentrations ranging from 0.4 to 5 mM. β-galactosidase activity was assayed after a 1-hour stress exposure. For the Yap1p-dependent promoter using the YRE_TRX2_-CYC1_TATA_-lacZ construction, we did not observe any significant differences in β-galactosidase activity between strains T73 and T73*trx2∆* under either the YPD or YPG conditions (Figure 3C and 3E). However, when we analyzed the Yap1p-Skn7p-dependent promoter using the GCRE_GPX2_-CYC1_TATA_-lacZ construction, we observed a significant decrease in the β-galactosidase levels for strain T73*trx2∆* as compared with the control strain in both growth media. Nevertheless, differences were much greater in YPG medium (Figure 3D and 3F) due to the strong induction of the GCRE promoter caused by growth on glycerol in strain T73 (Figure S3). In order to validate the involvement of Trx2p in the promoter regulation by Skn7p, we did similar experiments using the laboratory BY4741 and BY4741*trx2∆* strains transformed with the above-described constructions to obtain identical results (Figure S4). All together, these results demonstrate that the absence of the *TRX2* gene negatively affects the activation of the Yap1p-Skn7p-dependent promoter, but not the Yap1p-dependent promoters, and they suggest a new relationship between Trx2p and Skn7p activity.

### The Skn7p-dependent heat shock gene expression under oxidative stress is affected by the absence of TRX2

**Figure 4:**
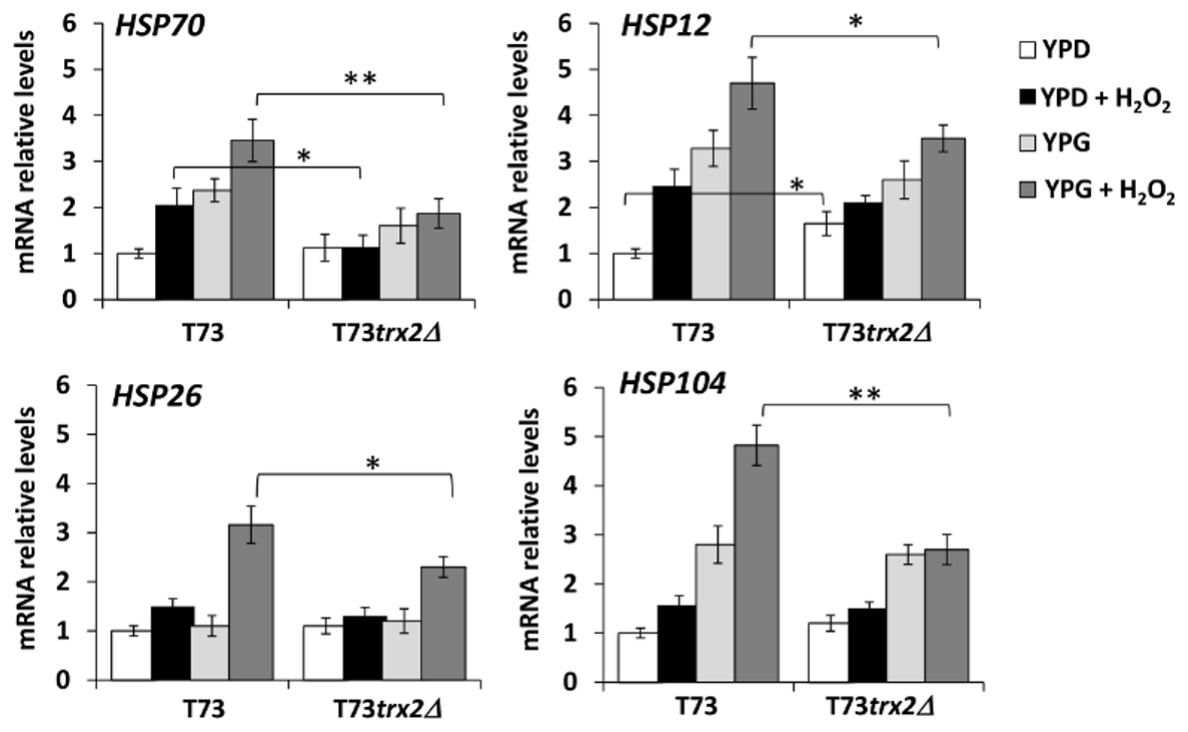
The expression analysis of the heat shock protein genes after exposure to oxidative stress conditions. (A) Northern blot analysis of HSP12, HSP26, HSP70 and *HSP104* in strains T73 and T73*trx2∆* grown on YPD or YPG to the mid-log phase and treated with 5 mM H_2_O_2_ for 60 min. (B) Relative expression levels were quantified by an image analysis. The data for each gene were normalized to the expression levels of the control strain in YPD medium under non stressed conditions. Three independent experiments were carried and the error bars corresponding to SD are shown. Statistical analyses were performed using a Student´s t-test between samples in the same growth media, YPD or YPG (* p< 0.05, ** p< 0.01).

It has been described that Skn7p is required for the full induction of the heat shock gene expression by hydrogen peroxide since the *skn7∆* mutation significantly lowers the mRNA level for several heat shock proteins, such as Hsp12p, Hsp26p, Hsp70p and Hsp104p, thus aggravating oxidative stress [44]. In order to check whether *TRX2* gene deficiency affects Skn7p activity under oxidative stress or not, we analyzed the mRNA expression levels of *HSP70*, *HSP26*, *HSP12* and *HSP104* in the *TRX2* gene-modified wine strain under the same growth and stress conditions as in the previous experiments (Figure 4 and Figure S5). All the genes exhibited a significantly increased expression at 5 mM H_2_O_2_ in control strain T73, with the highest expression levels noted in YPG medium. In the T73*trx2∆* mutant, the expression level of *HSP70* was significantly reduced in both the growth media plus H_2_O_2_ if compared with the T73 control strain, while other heat shock proteins, like *HSP12*, *HSP26* and *HSP104*, also significantly decreased in the T73*trx2∆* mutant in YPG medium after H_2_O_2_ exposure.

These results indicate that the absence of the *TRX2* gene negatively affects the induction of other Skn7-dependent genes under oxidative stress conditions, similarly to the phenotype of the *skn7∆* mutant [44]. Therefore, Trx2p may play a role in Skn7p activity regulation.

### Thioredoxin deficiency increases Skn7p phosphorylation under oxidative stress

It has been recently described that the Skn7p transcription factor is specifically phosphorylated under oxidative stress conditions, which facilitates and stabilizes its interaction with Yap1p during the antioxidant gene expression [23]. However, very little is known about the regulatory mechanism controlling Skn7p phosphorylation and transcriptional activation.

**Figure 5:**
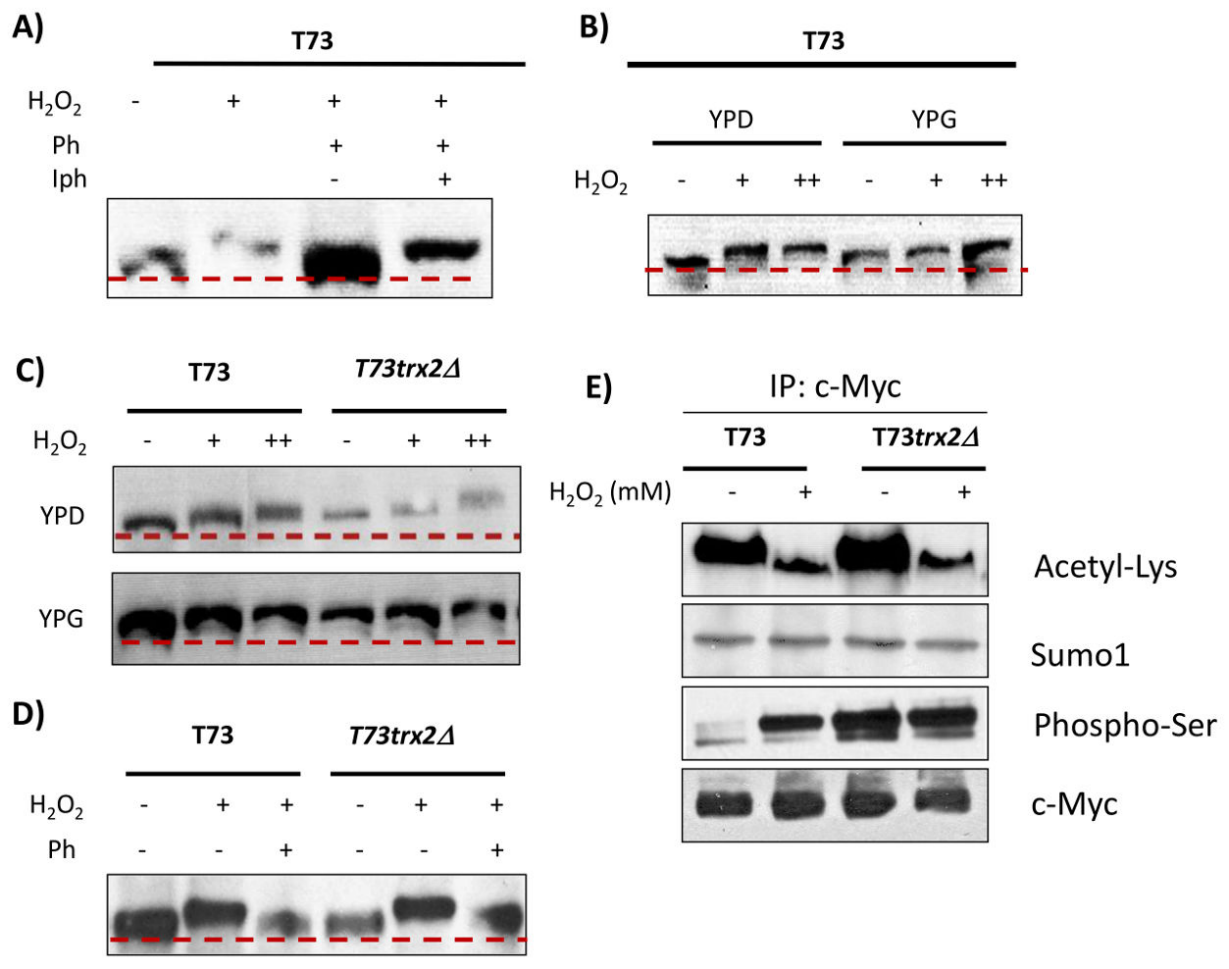
The Skn7p phosphorylation pattern after exposure to different H_2_O_2_ concentrations. (A) Cells from the T73-SKN7 (myc_13x_) control strain grown in YPD medium until the mid-log phase were exposed to 0.4 mM for 60 min. Lambda phosphatase (Ph) and phosphatase inhibitors (Iph) were added to the protein extracts as indicated and samples were incubated for 30 min at 30°C, following the manufacturer´s recommendations. Skn7p-myc western blotting was performed after protein separation in 8% SDS-PAGE. (B) The T73-SKN7 (myc_13x_) strain was cultivated in YPD and YPG medium until the mid-log phase and cells were exposed to 0 (-), 0.4 mM (+) and 5 mM (++) H_2_O_2_ for 1 h. (C and D) The Skn7p phosphorylation pattern in the TRX2 gene-modified strain under oxidative stress, 0.4 mM and 5 mM H_2_O_2_ for 60 min. (D) The protein extracts from the cells grown on YPD and exposed to 0.4 mM H_2_O_2_ for 1 h were treated with lambda-phosphatase, as indicated. (E) Analysis of the different post-transcriptional modifications using Skn7-Myc-tagged immunoprecipitation. Cells were cultivated in YPD medium and 0.4 mM H_2_O_2_ was added for 1 h. Representative experiments from five replicates are shown. A dashed gray line was added to the figure based on the migration of the untreated wild-type samples for each analyzed gel.

To investigate the putative role of thioredoxin II in Skn7p activity regulation, we analyzed the phosphorylation event on this transcription factor in strains T73 and T73*trx2∆*. We constructed a Skn7p-(myc_13x_) tagged variant by genome integration to analyze Skn7p phosphorylation at low and high H_2_O_2_ doses in strains T73 and T73*trx2∆* (Figure 5). The loading controls for the gels in Figure 5 are shown in Figure S6. We initially confirmed Skn7p phosphorylation by observing an electrophoretic shift in the Skn7p band after 0.4 mM hydrogen peroxide treatment in the T73-SKN7(myc_13x_) strain (Figure 5A), as previously described for laboratory yeast [23]. The Skn7p band displayed a poorer mobility shift when samples were treated with 100 U of λ phosphatase for 30 min at 30°C. The protein shift observed at 0.4 mM H_2_O_2_ was maintained when the sample was treated with λ phosphatase in the presence of phosphatase inhibitors (Figure 5A). All together, these findings demonstrate that the Skn7p electrophoretic mobility shift corresponds to protein phosphorylation. A similar Skn7p phosphorylation pattern was observed between YPD and YPG medium for the T73 strain using 0.4 and 5 mM H_2_O_2_ (Figure 5B). Yet the Skn7p band corresponding to the cells growing in glycerol without H_2_O_2_ treatment was already retarded if compared to the growth on glucose, which is likely due to endogenous ROS generation by a respiratory metabolism that increases the phosphorylation state of Skn7p as a result of stressful conditions. Despite this phenomenon, Skn7p phosphorylation increased in both growth media when increasing the H_2_O_2_ dose, although phosphorylation was higher in YPG than in YPD. 

Figure 5C shows the Skn7p phosphorylation state at different H_2_O_2_ doses in strains T73 and T73*trx2∆* in YPD and YPG media. As seen in the representative experiment of three independent replicates, the electrophoretic shift of Skn7p was greater in the T73*trx2∆* mutant than in the control strain, especially in the presence of 5 mM H_2_O_2_ in both growth media. We also observed that Skn7p showed retarded migration in the *trx2∆* mutant and that it grew in both media without H_2_O_2_ treatment when compared with the T73 wild type, which is likely due to a higher level of endogenous oxidative stress as a result of thioredoxin II being absent. These results indicate that the Skn7p phosphorylation state might be affected by both the fermentative or respiratory metabolism and by Trx2p, thus altering the antioxidant transcriptional response. Figure 5D illustrates increased Skn7p mobility after λ phosphatase treatment in both strains, indicating that the Skn7p mobility shift in the modified strain was due, at least in part, to protein phosphorylation. To better demonstrate that the altered Skn7p electrophoretic mobility in the T73*trx2∆* mutant was due to phosphorylation events, we immunoprecipitated the Skn7 13xMyc-tagged protein using anti-Myc agarose beads and probed for several post-translational modifications (PTMs) (Figure 5E). The anti-acetyl-lysine blot showed that the samples treated with H_2_O_2_ decreased Skn7p acetyl-lysine levels, suggesting a possible role in Skn7p activation, but no differences were observed between the T73 and T73*trx2∆* mutant strains. Another PTM that can affect electrophoretic mobility is sumoylation, which has been shown to play important roles in protein activity and stability. However no differences were observed in the sumoylation state between the analyzed samples. Finally we probed for the phosphor-serine levels and we observed that the T73*trx2∆* mutant showed higher Ser phosphorylation levels than the control strain.

All these results suggest that phosphorylation, and possibly other post-translational modifications still to be tested, regulate Skn7p activity under oxidative stress caused by exogenous H_2_O_2_ addition, and that thioredoxins modulate this phenomenon. These regulatory modifications in Skn7p are also dependent on the fermentative or respiratory metabolic state, which reinforces the relevance of this transcriptional factor under different oxidative stresses. In addition, these results also indicate an inverse correlation between the increased Skn7p phosphorylation levels in the T73*trx2∆* mutant and the lower expression of the Yap1p-Skn7p-regulated antioxidant genes.

## Discussion

Wine *Saccharomyces cerevisiae* strains, adapted to anaerobic must fermentations, undergo oxidative stress when grown under aerobic conditions, especially during biomass propagation in the industrial active dry yeast (ADY) production process [28,45]. The problem lies in that the oxidative metabolism of sugars favors high biomass yields, but also causes increased oxidative damage of cell components. The importance of the *TRX2* gene in the oxidative stress response under industrial conditions has been evidenced by the improved phenotype of a wine yeast strain overexpressing the *TRX2* gene [7,29]. Thus, the comprehension of the regulatory mechanisms controlling the oxidative stress response in wine yeasts under aerobic conditions can be used as a potential biotechnology improvement tool.

In this study, we have analyzed the biochemical, transcriptional and post-translational effects of *TRX2* gene deficiency on the oxidative stress response, especially at high H_2_O_2_ doses, and its dependence on the different carbon source media in natural wine yeasts. Our results reveal that wine yeasts are more resistant to several oxidant compounds under fermenting conditions than under respiratory conditions, which contrasts with previous data for lab yeasts [46,47]. These results can be explained by the ¨domestication¨ and adaptation of wine yeast to the fermentative metabolism of sugars over centuries, which has made them more resistant to different stresses under fermenting conditions [45,48]. Thus the specific study of the oxidative stress response in industrial yeasts is necessary. 

We observe that the absence of Trx2p causes growth defects only under respiratory metabolism, showing increased sensitivity to H_2_O_2_ under these conditions. Studies using glucose as a carbon source with the individual and double mutants of thioredoxins have shown that Trx2p accordingly plays a more important role than Trx1p, which indicates that the two thioredoxins do not perform overlapping functions exactly [6,49]. Our results demonstrate that Trx2p is essential only for the wine yeasts growing on glycerol. This effect has also been observed for other antioxidant defenses which are significantly influenced by the growth medium used for the experiments done [47]. When we analyzed different redox parameters, unexpectedly we did not observe any significant differences in either lipid peroxidation or the GSH/GSSG ratio, as previously shown for the W303*trx2∆* lab strain [5], but we noted lower catalase levels in YPG after hydrogen peroxide exposure, which can be related directly to growth defects and stress sensitivity [35]. 

Although the biochemical parameters did not undergo major modifications in the T73*trx2∆* strain, we searched for a differential antioxidant transcriptional response based on our previous work. We show that *TRX2* deficiency alters several oxidative stress-related genes under industrial propagation conditions [15]. As thioredoxins have been postulated to be the negative regulators of the Yap1p function [8,9], we went on to analyze the expression levels of several oxidative stress-related genes regulated by Yap1p, as well as others regulated by the cooperation between Yap1p and Skn7p [21,22]. We selected these genes to correlate variations in their expression levels with increased stress sensitivity under aerobic conditions. Laboratory strains are genetically very distant to any other *S. cerevisiae* strains [41,42], and this genetic distance and the complexity of Yap1p and Skn7p gene regulation suggest the need to study other *S. cerevisiae* strains from different sources to completely understand oxidative stress regulation. 

The expression analyses in the T73 control strain showed that only the genes belonging to the thioredoxin system (*TRX1, TRX2* and *TRR1*) are significantly induced under 5 mM H_2_O_2_ under high glucose conditions, whereas a broader and more intense response in several genes is produced by this same treatment under respiratory growth conditions. Interestingly when we analyzed the antioxidant gene expression levels for the T73*trx2∆* strain in YPD and YPG medium, we generally observed significant differences in gene expression only under respiratory conditions. We noted the up-regulation of the Yap1p- dependent genes after hydrogen peroxide treatment, which correlates with the Yap1 negative regulation by thioredoxins [8]. We also saw a down-regulation of the antioxidant genes regulated by the cooperation between Yap1p and Skn7p (such as *GSH1*, *TSA1* and *TRR1*) and putative Yap1p and Skn7p-dependent gene *GRX5* (www.yeastract.com). These results suggest a relationship between Trx2p and the Skn7p transcription factor, which are supported by the fact that although a W303*skn7∆* mutant does not show increased *TRX2* gene expression after hydrogen peroxide exposure, it does in a *yap1∆* mutant [50]. 

The analysis of the Yap1p and Skn7p-dependent promoters regulation by β-galactosidase assays clearly demonstrates Skn7p-dependent promoter activation is affected by the *TRX2* gene in wine strains. In contrast, the Yap1p-dependent promoter activation by the β-galactosidase assays does not vary in the absence of *TRX2* in natural yeasts, but the Yap1p-dependent genes are up-regulated by the Northern blot experiments. These results suggest another putative mechanism to activate the oxidative stress response in *S. cerevisiae* at a high H_2_O_2_ dose, one that is not exclusively related to Yap1p. This hypothesis is also supported by the fact that the activated expression of *TRX2* is observed under oxidative stress conditions in a *skn7∆yap1∆* mutant [21], suggesting the existence of another minor induction mechanism. 

The involvement of Trx2p in the regulation of Skn7p transcription factor activity is reinforced by the negative effect of *TRX2* deletion on the expression of several heat shock protein genes like *HSP70*, *HSP12*, *HSP26* and *HSP104*. They are induced mainly by the Heat Shock Factor (Hsf1p) under heat shock stress, but are regulated by Skn7p under oxidative stress conditions [44]. Our T73*trx2∆* strain data demonstrate similar defects on the HSPs expression levels after oxidative stress to those observed in a *skn7∆* mutant [44]. 

We finally demonstrate that Trx2p regulates Skn7p phosphorylation events, and therefore modulates Skn7p-dependent promoter activation. It has been recently demonstrated that the Ser/Thr phosphorylation of Skn7p is required for the antioxidant gene activation and strong interaction with Yap1p [23,24], which is also phosphorylated inside the nucleus [10]. In fact, Skn7p phosphorylation requires the presence of Yap1p because a *yap1∆* mutant does not show oxidant-dependent Skn7p phosphorylation [23]. In addition, it has been postulated that Skn7p can be regulated by the Ras/PKA and MAPK pathway under oxidative stress conditions [51]. However, very little is known about the components involved in oxidant-dependent Skn7p phosphorylation. We report that transcription factor Skn7p is hyperphosphorylated in the T73*trx2∆* strain after H_2_O_2_ exposure. Hyperphosphorylation might cause Skn7p inactivation as the down-regulation of Yap1p-Skn7p-regulated genes and no activation of the Skn7p-dependent promoter in β-galactosidase assays were observed for the T73*trx2∆* mutant. There are several putative residues in Skn7p than can participate in phosphorylation activation, such as T437, T449, I428 and V429 [23], and it is possible that different phosphorylation states of important residues can positively or negatively regulate Skn7p activity. Therefore, it is feasible that Trx2p can indirectly modulate the Skn7p phosphorylation state by regulating other components, like the kinases/phosphatases involved in the MAPK cascade and oxidative stress response. Interestingly, it has been described that thioredoxin modulates mammalian *ASK1* kinase activity by non-covalent interactions to then participate in the regulation of the apoptosis signal [52]. Further evidence that thioredoxin regulates other protein activities is that *TRX2* manipulation directly affects PAPS [13], ribonucleotide reductase [14] and hexokinase II [15]. 

All together, these results match a tentative regulatory model where Trx2p acts not only via its oxidoreductase activity on Yap1p in regulating the response to hydrogen peroxide dose, but it can also be a novel Skn7p modulator by regulating its phosphorylation state. Thus, Trx2p may perform dual functions in controlling oxidative stress response by negatively regulating Yap1p and positively modulating Skn7p activity. However, very little is known about the complex molecular response to oxidative stress in yeast, and Skn7p activity regulation by Trx2p is a mechanism that merits further research.

## Conclusions

This study demonstrates the involvement of Trx2p in oxidative stress response regulation on wine yeasts under respiring conditions. *TRX2* gene deficiency increases H_2_O_2_ sensitivity, causes the down-regulation of the Yap1p-Skn7p-dependent genes and produces Skn7p hyperphosphorylation. Based on these results, we propose Trx2p to be a novel regulator of the oxidant-dependent Skn7p phosphorylation by hydrogen peroxide in wine yeasts.

## Supporting Information

Figure S1 **The antioxidant genes in the wild-type T73- and *TRX2-*modified strains exposed to oxidative stress.**Northern blot analysis of the RNA isolated by the hot acid method from yeast strains T73 and T73*trx2∆* grown on YPD or YPG to the mid-log phase, and treated for 60 min with 5 mM H_2_O_2_. The Yap1p and the Yap1p-Skn7p-dependent gene groups were formed following the previously described information [21, 22, 43] and from the Yeastract web site (http://www.yeastract.com). Three independent experiments were carried out for each analyzed gene and one representative experiment is shown.
(TIF)

Figure S2 **mRNA expression levels of *GLR1*, *GTT1* and *TRX1* by qPCR analysis in YPD and YPG medium and under the presence of 5 mM H_2_O_2_ for 1 h.** Expression levels were normalized using actin gene expression as control and normalized to control conditions in YPD medium. One representative experiment was analyzed by qPCR using three technical replicates.
(TIF)

Figure S3 **(**A**) *GPX2* gene expression analysis by Northern blot experiments in the T73 and T73*trx2∆* grown on YPD or YPG to the mid-log phase, and treated for 60 min with 5 mM H_2_O_2_.** (B) mRNA quantification by an image analysis from one representative experiment.
(TIF)

Figure S4 **Promoter regulation by the Yap1p and Skn7p transcriptional factors in strains BY and BY*trx2∆*.** (A) The cells carrying the depicted modification of the *TRX2* promoter fused to the LacZ vector construction (BY-YRE and BY*trx2∆*-YRE) for Yap1p recognition or (B) the cells carrying the depicted modification of the GPX2 promoter fused to the LacZ vector construction (BY-GCRE and by BY*trx2∆*-GCRE) for Yap1p-Skn7p recognition were cultured in YPD (C, E) or YPG medium (D, F) until the mid-log phase and were treated with different H_2_O_2_ concentrations (0.4, 1, 2.5 and 5 mM) for 60 min. Three independent experiments were carried out and the error bars corresponding to SD are shown.
(TIF)

Figure S5 **The HSPs genes in the wild-type T73- and *TRX2-*modified strains exposed to oxidative stress.** Northern blot analysis of the RNA isolated by the hot acid method from yeast strains T73 and T73*trx2∆* grown on YPD or YPG to the mid-log phase, and treated for 60 min with 5 mM H_2_O_2_. One representative experiment from three independent experiments is shown.
(TIF)

Figure S6 **Loading controls from Figure 5.** Membranes were stained with Coomassie blue to demonstrate a similar migration pattern in the total protein load.
(TIF)

Table S1 **Genes and primers used for the amplification of DNA probes.**
(DOC)
