# Supplementary figures and images for: Trx2p-dependent Regulation of Saccharomyces cerevisiae Oxidative Stress Response by the Skn7p Transcription Factor under Respiring Conditions
Source: PLoS One. 2013 Dec 23;8(12):e85404. doi: 10.1371/journal.pone.0085404 (PMC3871606; doi:10.1371/journal.pone.0085404)

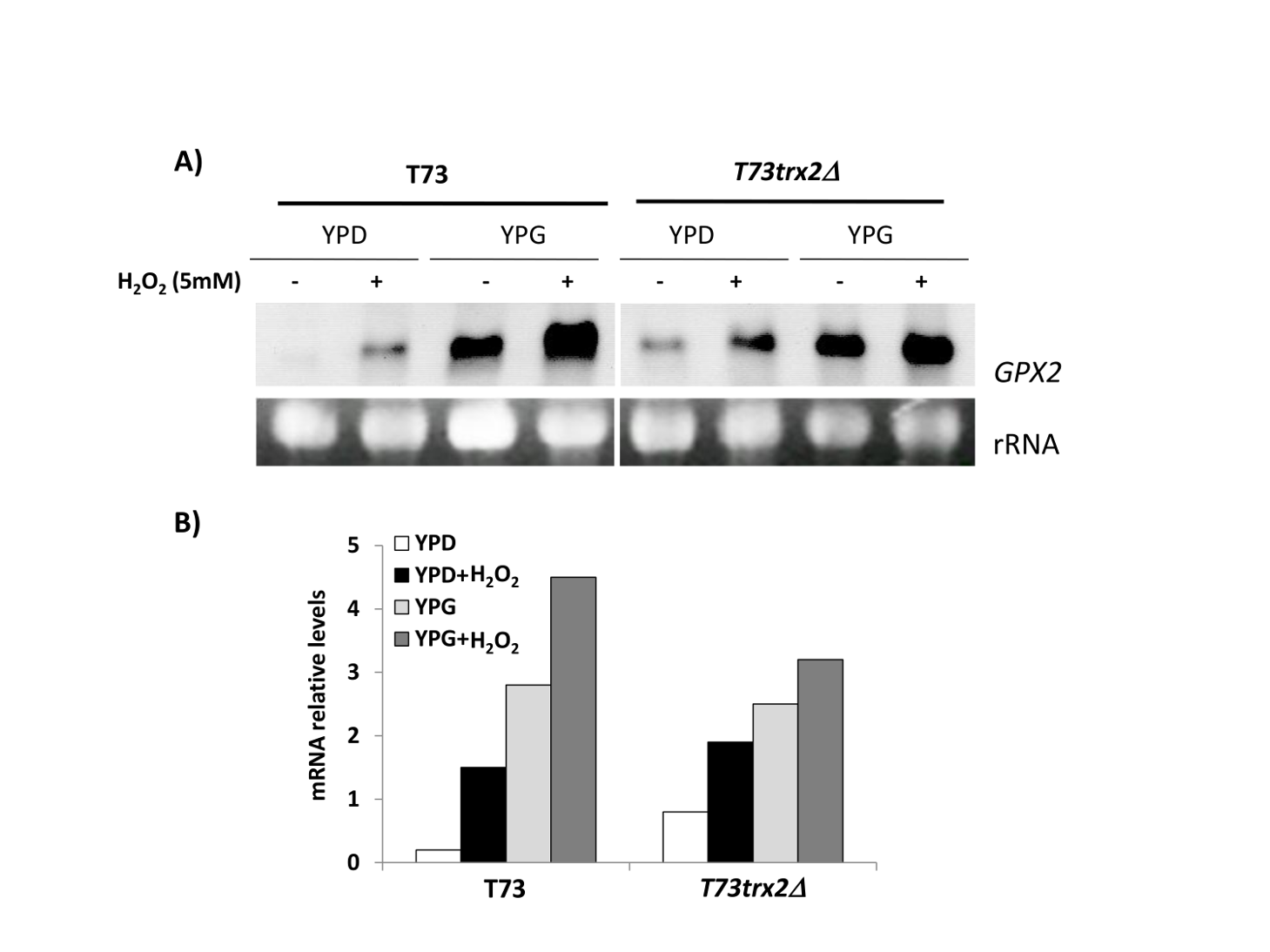

Supplement: Figure S3 — (A) GPX2 gene expression analysis by Northern blot experiments in the T73 and T73trx2∆ grown on YPD or YPG to the mid-log phase, and treated for 60 min with 5 mM H2O2. (B) mRNA quantification by an image analysis from one representative experiment. (TIF) [file pone.0085404.s003.tif]
