# Supplementary material for: Trx2p-dependent Regulation of Saccharomyces cerevisiae Oxidative Stress Response by the Skn7p Transcription Factor under Respiring Conditions
Source: PLoS One. 2013 Dec 23;8(12):e85404. doi: 10.1371/journal.pone.0085404 (PMC3871606; doi:10.1371/journal.pone.0085404)
Supplement: Table S1 — Genes and primers used for the amplification of DNA probes. (DOC) [file pone.0085404.s007.doc]

| **Supplementary Table 1.** Genes and primers used for the amplification of DNA probes | | | |  |
| --- | --- | --- | --- | --- |
| **Probe** | **Primer** | **Sequence (5´- 3´)** | **Length (bp)** | |
|  |  |  |  | |
|  | ***GSH1-1*** | **CCCGATGAAGTCATTAACA** |  | |
| ***GSH1*** |  |  | **945** | |
|  | ***GSH1-2*** | **GGAAAAGGTCAAAATGCT** |  | |
|  |  |  |  | |
|  | ***GLR1-1*** | **GAGCTGCATCTTATGGTGC** |  | |
| ***GLR1*** |  |  | **729** | |
|  | ***GLR1-2*** | **CGATGGCTTTGAGTCATCT** |  | |
|  |  |  |  | |
|  | ***GTT1-1*** | **GATGCTAACTTCCGTGCTC** |  | |
| ***GTT1*** |  |  | **530** | |
|  | ***GTT1-2*** | **GCTCAACTTCCCATCAACC** |  | |
|  |  |  |  | |
|  | ***GRX5-1*** | **CATAAGGTCATTTTCCCCC** |  | |
| ***GRX5*** |  |  | **407** | |
|  | ***GRX5-2*** | **CTTCTTCAGGTACCAATGCC** |  | |
|  |  |  |  | |
|  | ***TSA1-1*** | **CAAGTTCAAAAGCAAGCTCC** |  | |
| ***TSA1*** |  |  | **461** | |
|  | ***TSA1-2*** | **TCAACCAATCTCAAGGCTTC** |  | |
|  |  |  |  | |
|  | ***TRR1-1*** | **ATGAAGGTATGATGGCGAAC** |  | |
| ***TRR1*** |  |  | **779** | |
|  | ***TRR1-2*** | **ATCCTGAACATCACCAGCAG** |  | |
|  |  |  |  | |
|  | ***TRX2-1*** | **AAATCCGCTTCTGAATAC** |  | |
| ***TRX2*** |  |  | **300** | |
|  | ***TRX2-2*** | **CTATACGTTGGAAGCAATAG** |  | |
|  |  |  |  | |
|  | ***TRX1-1*** | **GCCGACAGTCTGTTGAATTG** |  | |
| ***TRX1*** |  |  | **726** | |
|  | ***TRX1-2*** | **GTTTCCTCCACCTCCTTTG** |  | |
|  |  |  |  | |
|  | ***SOD2-1*** | **TGTGCCGAAAAATTTCCGCG** |  | |
| ***SOD2*** |  |  | **410** | |
|  | ***SOD2-1*** | **TTGGCCAGAAGATCTGAGAG** |  | |
|  |  |  |  | |
|  | ***CTT1-1*** | **ATGCCAATAAGATCAATCAGC** |  | |
| ***CTT1*** |  |  | **1715** | |
|  | ***CTT1-2*** | **TTGGCACTTGCAATGGACC** |  | |
|  |  |  |  | |
|  | ***HSP12-1*** | **ATGTCTGACGCAGGTAGAAA** |  | |
| ***HSP12*** |  |  | **330** | |
|  | ***HSP12-2*** | **TTACTTCTTGGTTGGGTCTT** |  | |
|  |  |  |  | |
|  | ***HSP26-1*** | **GCTGGCGCTCTTTATGATCC** |  | |
| ***HSP26*** |  |  | **300** | |
|  | ***HSP26-2*** | **GAACTTACCAGAGCTGCTCTCC** |  | |
|  |  |  |  | |
|  | ***HSP70-1*** | **GCTGGTTTGAATGTCTTGCG** |  | |
| ***HSP70*** |  |  | **435** | |
|  | ***HSP70-2*** | **AATTCTGGTAGAACCACCGACC** |  | |
|  |  |  |  | |
|  | ***HSP104-1*** | **GGACGACGCTGCTAACATC** |  | |
| ***HSP104*** |  |  | **225** | |
|  | ***HSP104-2*** | **TCCAGAATCCTTACACCATG** |  | |
